# Removing Craniofacial Titanium Screws: Technical Note

**DOI:** 10.7759/cureus.19891

**Published:** 2021-11-25

**Authors:** Jaims Lim, Amade Bregy, Kevin Gibbons

**Affiliations:** 1 Neurological Surgery, Buffalo General Medical Center, Buffalo, USA

**Keywords:** craniofacial trauma, titanium cranioplasty, titanium 2-d miniplates, titanium 3-d miniplates, open craniotomy

## Abstract

Craniotomy, cranioplasty, and craniofacial procedures may involve reoperation for additional treatment of the primary pathological condition or treatment of complications, requiring removal of previously placed hardware. During removal of the titanium hardware, there is a risk of losing, dropping, or misplacing the titanium screws because of their small size. There are also instances of difficulty disengaging the screw from the screwdriver. We describe the use of a plastic specimen cup in retrieving titanium screws after detaching them from the screwdriver by screwing the screw into the cup, thus rapidly and safely securing and storing screws for collection/discarding or possible reuse. When the empty screwdriver is used to retrieve and unscrew the titanium screw from the cranial flap or the skull bone, a plastic specimen cup should be placed adjacent to the site of screw removal. Once the screw is removed, while it is still fastened to the screwdriver, it is immediately re-screwed and secured onto the base of the plastic specimen cup, which is then placed into a second plastic specimen cup. This method prevents misplacement or dropping of the screw that may otherwise occur when manipulating the screw on or off the screwdriver and avoids perforating the surgeon’s glove during handling. We describe the adjunctive use of a plastic specimen cup when removing cranial screws and hardware to rapidly and safely detach the screw and prevent the misplacement, dropping, or loss of screws intraoperatively that results in additional operative time and personnel assistance.

## Introduction

Cranioplasty procedures are associated with reoperation and complication rates ranging from 2% to 50% [1-5]. Recurrent pathology or complications such as infections, poor cosmesis, or hematomas may warrant a reoperation that requires removal of the cranial flap along with hardware and titanium screws [1,2,6]. Although no formal data have ever been published, during removal of the screws, there is a risk of misplaced, dropped, or lost screws intraoperatively while the screw is being removed from the skull fixation, handed off to the surgical technician assisting the case, and most often while disengaging the screw from the cranial-plating screwdriver. This calls for additional intraoperative fluoroscopic imaging, instrument counting, and time loss while trying to locate the misplaced screw. We present a simple method of retrieving the titanium cranioplasty screw and safely returning it to the operative table for collection and possible reuse.

## Technical report

Rationale

When a cranioplasty or craniofacial hardware revision takes place, the titanium or metal hardware screws need to be removed to subsequently remove the securing plates and ultimately the cranioplasty flap or bone. The first step to this procedure is removing the titanium screws; given a non-resorbable neuro-plating system was utilized [6]. From the moment an empty cranial plating screwdriver is utilized to unscrew/remove the screw from the bone and to transfer it back to the surgical technician, the screw may drop out of place from the screwdriver or it may be mishandled by the surgical technician and lost while trying to detach the screw and move it to a collection tray on the operative back table. There is a risk of glove perforation while detaching screws from the screwdriver. There have been no quotes of the prevalence of the aforementioned complications, but if the screw is misplaced, depending on the institutional policy, it may warrant further fluoroscopic imaging of the cranium as well as time expended in attempts to locate the screw. This results in increased use of operative resources and personnel as well as prolonging the patient’s anesthesia time.

Method

**Figure 1:**
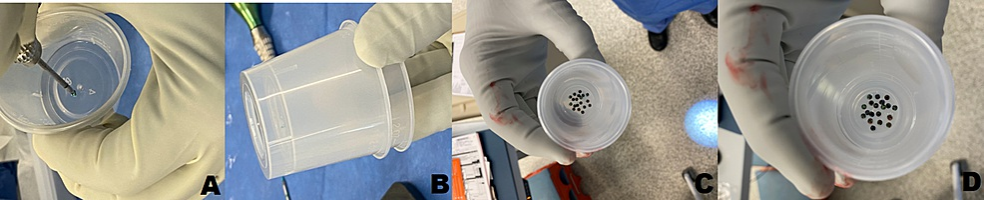
Securing of titanium screws into the plastic specimen cup. Photograph of secured fastening of removed 4 mm titanium screws to the base of a 4 oz sterile surgical plastic specimen cup (A) and placement of cup in a second cup to prevent perforation of OR table drape or accidental injury (B) is shown during a cranioplasty revision secondary to extrusion of hardware through the skin. The screws can be safely positioned and also be reloaded for re-use. Multiple screws can be placed into the same specimen container (C and D).

We present the simple use of a sterile plastic collection cup to be positioned next to the open cranial wound prior to the removal of screws to prevent the complication of lost or misplaced neuro-plating screws. When the screw is removed from its secure position within the cranial flap or the skull, the screw, while attached to the screwdriver, is immediately then screwed into the base of the plastic specimen cup (Figure 1A), disengaged from the driver, and the cup is then placed into a second cup (Figure 1B) to prevent any injury due to the sharp nature of the screw or any perforation of gloves or drapes. This prevents miscounts of screws removed as well as provides clear segregation of removed screws as opposed to placing them back into the native screw holder. Multiple screws can be placed in this fashion into the same specimen container (Figures 1C, 1D). This technique allows the rapid and secure collection and fixation of the screw into a collection container and reduces the risk of accidental loss; it also allows the screws to be reloaded onto an empty screwdriver for possible reuse. Further, keeping all screws secured and fastened to the specimen cup aids and facilitates the counting of screws at the conclusion of the case.

## Discussion

The use of titanium hardware in securing bone and cranioplasty flaps to the native skull was initially described in the 1990s [7,8]. Titanium plates and screws have been shown to be a cost-effective and safe option for correcting craniofacial abnormalities, replacing prosthetic and native bone flaps to the surrounding skull [9]. Comparison of 392 craniectomy patients undergoing cranioplasty demonstrated the use of titanium hardware was associated with decreased complication rates compared to bone clamps and sutures [10]. Studies have also compared the use of titanium cranial plates and screws versus other hardware, including metal wires and bone cement, and have found that titanium screws and plates result in more secure fixation compared to other agents including hydroxyapatite [11,12].

Cranioplasty procedures involving removal and replacement of bone carry a complication rate of 2-50% [1,2]. Complications include palpability of the underlying hardware, infection, pain, hardware failure, and overlying skin erosion [6]. Many strategies and techniques regarding the salvage of infected bone flaps during craniectomies have been described [13,14], there is a lack of description regarding complications associated with the misplacement and retainment of cranial screws and hardware during the operation.

With the current widespread use of titanium craniofacial plates and screws for cranial procedures, there is a risk of losing or misplacing the titanium screws during the initial surgery or when patients need to undergo revision or redo surgery. As mentioned, this may result in prolonging the surgery and anesthesia time for the patient as well as additional radiation exposure to the patient if intraoperative skull fluoroscopy is needed to exclude misplacement of the hardware intracranially. To best avoid such complications and danger to the patient, we provided a simple method to rapidly collect and secure titanium screws when removed from the skull during cases such as cranial revision surgery. By collecting the screws in a plastic specimen cup, not only can they be reused but they can also be easily counted by the surgical technician at the end of the case, which could also aid in the closure of the case.

## Conclusions

During cranioplasty or craniofacial hardware revision, titanium or metal hardware screws need to be removed to remove the underlying plate, cranioplasty flap, or bone, and from the time the screw is removed to transfer to the surgical technician, the screw may drop out of place or it may be mishandled. We describe the adjunctive use of a plastic specimen cup when removing cranial screws and hardware to prevent the misplacement, dropping, and loss of screws intraoperatively, which also provides an opportunity for screws to be reloaded to an empty screwdriver for possible reuse. This method prevents the loss of screws intraoperatively after removal in hopes to save anesthesia and surgery time and prevent complications by avoiding misplacement of hardware intracranially.
